# Supplementary figures and images for: In Vitro Functionality and Endurance of GMP-Compliant Point-of-Care BCMA.CAR-T Cells at Different Timepoints of Cryopreservation
Source: Int J Mol Sci. 2024 Jan 23;25(3):1394. doi: 10.3390/ijms25031394 (PMC10855166; doi:10.3390/ijms25031394)

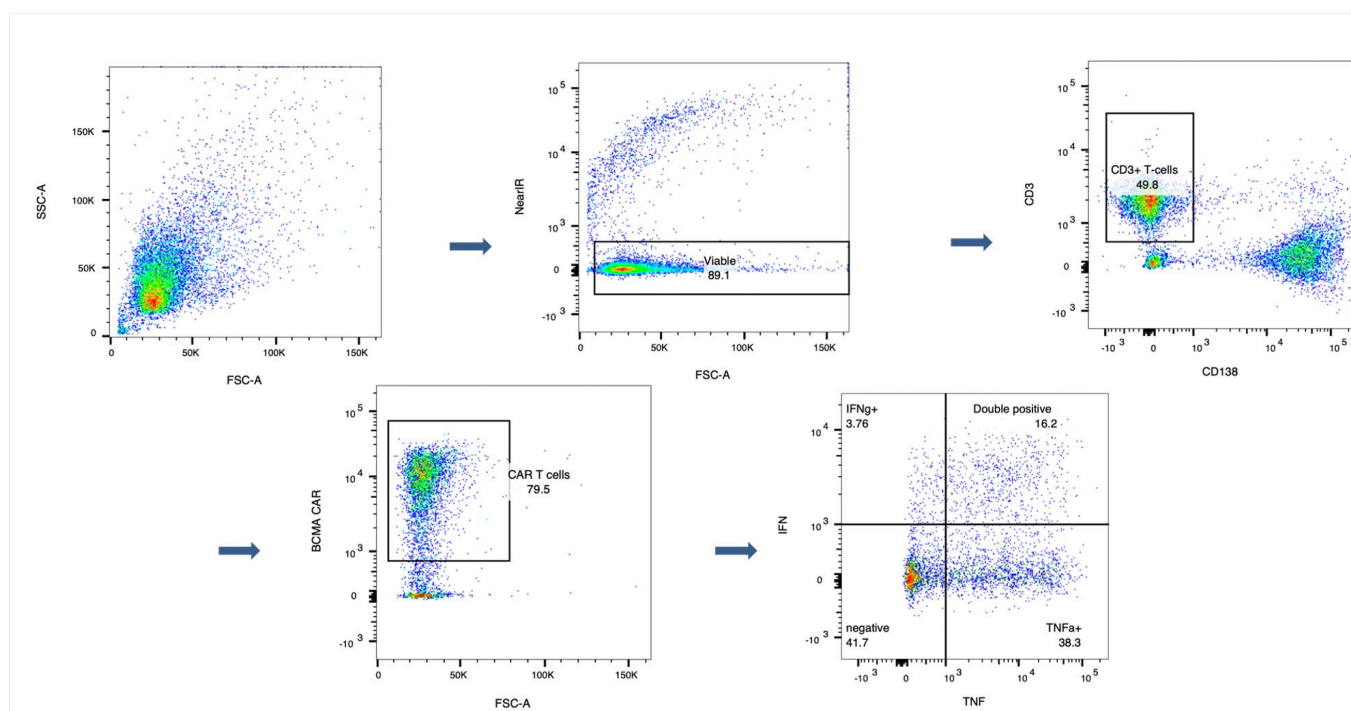

**Figure S1.** Gating strategy of ICS for detecting TNF- $\alpha$ - and IFN- $\gamma$ - producing cells.

Supplement: Supplementary file 1 [file ijms-25-01394-s001.zip › ijms-2792479-supplementary.pdf]
